# Efficacy of zinc glycinate reducing zinc oxide on intestinal health and growth of nursery pigs challenged with F18^+^ Escherichia coli

**DOI:** 10.1093/jas/skad035

**Published:** 2023-01-28

**Authors:** Ki Beom Jang, Vitor Hugo C Moita, Nicolas Martinez, Adebayo Sokale, Sung Woo Kim

**Affiliations:** Department of Animal Science, North Carolina State University, Raleigh, NC 27695, USA; Department of Animal Science, North Carolina State University, Raleigh, NC 27695, USA; BASF Corporation, Florham Park, NJ, 07932, USA; BASF Corporation, Florham Park, NJ, 07932, USA; Department of Animal Science, North Carolina State University, Raleigh, NC 27695, USA

**Keywords:** *Escherichia coli*, intestinal health, nursery pigs, zinc glycinate, zinc oxide

## Abstract

The objective of this study was to investigate effects of zinc glycinate (ZnGly) supplementation reducing zinc oxide (ZnO) in feeds on intestinal health and growth of nursery pigs challenged with F18^+^*Escherichia coli (E. coli).* In total, 72 nursery pigs (BW 6.5 ± 0.5 kg) were allotted in a randomized complete block design to nine treatments: (1) NC: no challenge/no supplement; (2) PC: *E. coli* challenge/no-supplement; (3) *E. coli* challenge/ZnO at 2,500 mg/kg; (4, 5, and 6) *E. coli* challenge/ZnGly at 400, 800, and 1,200 mg/kg; and (7, 8, and 9) *E. coli* challenge/ZnGly at 400 mg/kg and ZnO at 700, 1,400, and 2,357 mg/kg. Pigs were fed for 28 d based on two phases (phase 1: 14 d and phase 2: 14 d). On day 7, challenged groups were orally inoculated with F18^+^*E. coli* at 6 × 10^9^ CFU/mL whereas NC received saline solution. The PC showed reduced ADG (*P* = 0.076) and G:F (*P* = 0.055) during phase 1 and increased fecal score (*P* < 0.05) during the first week of postchallenge when compared with NC, whereas supplementation of ZnGly from 0 to 1,200 mg/kg linearly increased (*P =* 0.092) G:F and decreased (*P <* 0.05) the fecal score of the pigs challenged with F18^+^*E. coli*. Supplementation of ZnGly from 0 to 1,200 mg/kg had quadratic effects on TNF-α (*P =* 0.065; minimum 1.13 pg/mg at 850 mg/kg ZnGly), IL-8 (*P =* 0.093; minimum 0.53 ng/mg at 494 mg/kg), and protein carbonyl (*P =* 0.054; minimum 2.30 pg/mg at 675 mg/kg) and linearly increased mRNA expressions of ZIP4 (*P =* 0.057) and ZnT5 (*P =* 0.075) in the jejunum of the pigs. Supplementation of ZnGly from 0 to 1,200 mg/kg linearly increased (*P <* 0.05) the relative abundance of Actinobacteria and had quadratic effects on Cyanobacteria (minimum 0.67% at 625 mg/kg ZnO) and Proteobacteria (maximum 45.6 g/d at 735 mg/kg) at the phylum level, with linearly decreased (*P* < 0.05) *Enterobacteriaceae* at the family level in the jejunal mucosa-associated microbiota of the pigs. There was no difference in growth performance during the overall period, although pigs fed with ZnO at 2,500 mg/kg had greater (*P <* 0.05) ADG than pigs fed with ZnGly at 400 mg/kg during the first week of the post challenge period. In conclusion, ZnGly could be an alternative to the pharmaceutical use of ZnO without negatively affecting the growth of nursery pigs by enhancing intestinal Zn absorption, reducing intestinal inflammation and oxidative stress, and providing positive changes in jejunal mucosa-associated microbiota.

## Teaser

Zinc glycinate could be an alternative Zn source to replace the pharmaceutical use of ZnO in nursery feeds by reducing the negative impacts of F18^+^*E. coli*^*+*^ on growth and intestinal health of nursery pigs.

## Introduction

Zinc (Zn) is one of the essential microminerals and serves as a cofactor for metalloenzymes responsible for various physiological processes involved in cell repair and division, as well as nutrient metabolism (McCall et al., 2000). Previous studies have also shown that Zn can protect enterocytes from stressors under challenging conditions negatively affecting physiological and immune status (Roselli et al., 2003; Cousins, 2010; Brugger and Windisch, 2019). In swine production, inorganic Zn sources have been broadly used in commercial diets to efficiently provide Zn for the growth and health of pigs. Swine producers have used levels exceeding the requirement of zinc oxide (ZnO) ranging from 2,000 to 3,000 mg/kg in feeds for therapeutic use as growth promoter with diarrhea prevention, even though the requirement is only about 100 mg/kg for nursery pigs (NRC, 2012). Previous studies have shown that ZnO can produce reactive oxygen species to induce oxidative stress in bacteria (Sawai et al., 1998; Dwivedi et al., 2014) possibly due to the fact that microbial respiratory enzymes, such as nicotinamide adenine dinucleotide (NADH) dehydrogenase, would be inhibited by interaction with Zn ions (Messner and Imlay, 1999; Yoshinobu et al., 2003). In regards to the use of ZnO in swine feeds, there are growing concerns that pharmaceutical use of ZnO would cause antimicrobial resistance, and environmental issues related to Zn excretion (Lynegaard et al., 2021; Szuba-Trznadel et al., 2021). According to legislation released in the European Union, a maximum of 150 mg/kg of Zn in the feeds is a legal limit for the use in animal nutrition (European Parliament and the Council of the European Union, 2003) and veterinary therapeutic products containing the pharmaceutical levels of ZnO were phased out in 2022.

The growth and health of nursery pigs are susceptible to the reduced use of antimicrobial compounds in the feeds because the pigs would have impaired intestinal barrier functions during the post weaning period (Jang and Kim, 2019; Zheng et al., 2021). The pigs do not have fully developed intestinal function for nutrient utilization or a defensive system protecting from pathogenic bacteria such as enterotoxigenic *E. coli* commonly associated with post weaning diarrhea (Rhouma et al., 2017; Duarte et al., 2020; Xu et al., 2022). In particular, enterotoxigenic *E. coli* is characterized as causing intestinal inflammation and oxidative stress by releasing enterotoxins and increasing adhesion to enterocytes (Nagy and Fekete, 2005; Sun and Kim, 2017; Duarte et al., 2020). Therefore, enterotoxigenic *E. coli* is currently one of the prominent harmful bacteria causing impaired growth and intestinal health with post weaning diarrhea of the pigs, leading to significant economic losses throughout production.

There have been increased efforts to investigate and find alternative Zn sources to reduce the pharmaceutical use of ZnO without negatively affecting the performance of pigs (Castillo et al., 2008; Wang et al., 2010; Shen et al., 2014; Satessa et al., 2020). In particular, various forms of organic Zn chelated with amino acids, polysaccharides, proteins, or organic acids, have shown positive impacts on growth and biological responses of pigs due to the high bioavailability of Zn rather than inorganic Zn sources (Mazzoni et al., 2010; Zhang et al., 2018; Skiba et al., 2022). Previous studies suggest that organic Zn sources could be partially or equally replaced to the high levels of ZnO supplementation on growth performance of nursery pigs through providing a higher bioavailability than inorganic sources (Case and Carlson, 2002). Among organic Zn sources, zinc glycinate (ZnGly) could also provide potential benefits to improve Zn bioavailability, growth, and intestinal function, and reduce Zn excretion of nursery pigs (Wang et al., 2010; Nitrayova et al., 2012). Chelation with glycine could slightly increase stability and electrical neutrality for Zn as opposed to other amino acid chelation (Zhang et al., 2017). Because glycine has the lowest molecular weight of all of the amino acids, its size favors the stability of the chelate compounds to protect from undesirable chemical reactions in the gastrointestinal tract that limit mineral utilization, resulting in high bioavailability of Zn (Kulkarni et al., 2011; Ashmead, 2001). Considering the biological functions of Zn as well as the biological efficacy of organic mineral, ZnGly could effectively enhance the intestinal health of nursery pigs by positively regulating immune and oxidative stress responses during the post weaning period, leading to a decrease in the use of ZnO in the feeds.

Therefore, it is hypothesized that supplementation of ZnGly can reduce the use of ZnO by positively affecting intestinal health and growth performance of nursery pigs challenged with F18^+^*E. coli*. The objective of this study was to investigate the effect of ZnGly supplementation reducing ZnO in feeds on growth performance and intestinal health of nursery pigs challenged with F18^+^*E. coli.*

## Materials and Methods

The procedure of this study was reviewed and approved by North Carolina State University Animal Care and Use Committee (Raleigh, NC). The experiment was conducted at the North Carolina State University Metabolism Educational Unit (Raleigh, NC).

### Experimental design, animals, and diets

A total of 72 newly weaned pigs (PIC Camborough × DNA 600; 36 barrows and 36 gilts) at 3 weeks of age with the initial body weight (BW) at 6.5 ± 0.5 kg, were selected from sows which were not vaccinated for or infected with enterotoxigenic *E. coli* as suggested by previous studies (Luise et al., 2019a; Duarte et al., 2020; Kim et al., 2021; Xu et al., 2022). The nursery pigs were randomly allotted to nine treatment groups based on randomized complete block design with BW (heavy and light) and sex (barrows and gilts) as blocks. Each treatment group had 8 replicates with one pig per pen. The treatment groups (1 to 9) were as follows 1) NC: pigs not-challenged with *E. coli*; 2) PC: pigs challenged with F18^+^*E. coli*; 3) pigs challenged with F18^+^*E. coli* and fed ZnO (Zinc Nacional, Monterrey, Mexico) at 2,500 mg/kg in the diet providing 1,813 mg/kg of Zn as Zn content in the ZnO; 4–6) pigs challenged with F18^+^*E. coli* and fed ZnGly (BASF Corp., Florham Park, NJ) at 400, 800, and 1,200 mg/kg in the diets providing 104, 208, and 312 mg/kg of Zn as Zn content in the ZnGly, respectively; 7–9) pigs challenged with F18^+^*E. coli* and fed ZnGly at 400 mg/kg and ZnO at 700, 1,400, and 2,357 mg/kg in the diets providing 612, 1,119, and 1,813 mg/kg of Zn, respectively. The pigs were provided with feeds and water ad libitum throughout the experiment. Room temperature was maintained at 30 °C in the first week and it was reduced by 2 °C per week. The experimental period was 28 d and was divided into two dietary phases; phase 1 (weaning to day 14) and phase 2 (days 14–28). Experimental diets for phase 1 (Table 1) and phase 2 (Table 2) were mixed at the North Carolina State University Feed Education Unit (Raleigh, NC). All diets were in mash form. Feed disappearance and BW were measured weekly to calculate average daily gain (ADG), average feed intake (ADFI), and feed efficiency (G:F).

**Table 1.** Composition of experimental diets for phase 1

Item	NC	PC	ZnGly 400 mg/kg				ZnGly 800 mg/kg	ZnGly 1,200 mg/kg	ZnO 2,500 mg/kg
			ZnO 0 mg/kg	ZnO 700 mg/kg	ZnO 1,400 mg/kg	ZnO 2,357 mg/kg			
Feedstuff, %									
Corn, yellow	36.95	36.95	37.19	37.12	37.05	36.95	37.15	37.11	36.98
Soybean meal, 48% CP	18.00	18.00	18.00	18.00	18.00	18.00	18.00	18.00	18.00
Whey permeate	24.00	24.00	24.00	24.00	24.00	24.00	24.00	24.00	24.00
Poultry meal	10.00	10.00	10.00	10.00	10.00	10.00	10.00	10.00	10.00
Blood plasma	3.50	3.50	3.50	3.50	3.50	3.50	3.50	3.50	3.50
Fish meal	3.50	3.50	3.50	3.50	3.50	3.50	3.50	3.50	3.50
Poultry fat	1.50	1.50	1.50	1.50	1.50	1.50	1.50	1.50	1.50
Limestone	0.43	0.43	0.43	0.43	0.43	0.43	0.43	0.43	0.43
L-Lys HCl	0.51	0.51	0.51	0.51	0.51	0.51	0.51	0.51	0.51
DL-Met	0.25	0.25	0.25	0.25	0.25	0.25	0.25	0.25	0.25
L-Thr	0.17	0.17	0.17	0.17	0.17	0.17	0.17	0.17	0.17
L-Trp	0.02	0.02	0.02	0.02	0.02	0.02	0.02	0.02	0.02
Formic acid	0.50	0.50	0.50	0.50	0.50	0.50	0.50	0.50	0.50
Salt	0.22	0.22	0.22	0.22	0.22	0.22	0.22	0.22	0.22
Vitamin premix^1^	0.03	0.03	0.03	0.03	0.03	0.03	0.03	0.03	0.03
Trace mineral premix^2^	0.14	0.14	0.14	0.14	0.14	0.14	0.14	0.14	0.14
Zinc oxide	--	--	--	0.07	0.14	0.24	--	--	0.25
Zinc glycinate	--	--	0.04	0.04	0.04	0.04	0.08	0.12	--
Total	100.00	100.00	100.00	100.00	100.00	100.00	100.00	100.00	100.00
Calculated composition									
DM, %	91.1	91.1	91.0	91.0	90.9	90.8	91.0	90.9	90.8
ME, kcal/kg	3,411	3,411	3,409	3,407	3,405	3,401	3,408	3,407	3,402
CP, %	24.5	24.5	24.5	24.5	24.5	24.5	24.5	24.5	24.5
SID^3^ Lys, %	1.50	1.50	1.50	1.50	1.50	1.50	1.50	1.50	1.50
Ca, %	0.85	0.85	0.85	0.85	0.85	0.85	0.85	0.85	0.85
STTD^4^ P, %	0.48	0.48	0.48	0.48	0.48	0.48	0.48	0.48	0.48
Zn, %	0.02	0.02	0.03	0.07	0.12	0.19	0.04	0.05	0.19
Analyzed composition									
DM, %	90.93	90.98	90.78	90.53	91.08	91.21	90.67	90.72	91.10
CP, %	23.10	23.49	23.78	23.40	23.91	23.92	23.87	23.37	23.76
Zn^5^, %	0.01	0.01	0.02	0.07	0.12	0.16	0.03	0.04	0.18
Formate^5^, %	0.29	0.32	0.32	0.31	0.32	0.34	0.31	0.30	0.28
pH	5.55	5.57	5.50	5.53	5.53	5.50	5.52	5.49	5.60

^1^The vitamin premix provided per kilogram of complete diet: 6,614 IU of vitamin A as vitamin A acetate, 992 IU of vitamin D_3_, 19.8 IU of vitamin E, 2.6 mg of vitamin K as menadione sodium bisulfate, 0.03 mg of vitamin B_12_, 4.6 mg of riboflavin, 18.5 mg of D-pantothenic acid as calcium panthonate, 26.5 mg of niacin, and 0.07 mg of biotin.

^2^The trace mineral premix provided per kilogram of complete diet: 30 mg of Mn as manganous oxide, 100 mg of Fe as ferrous sulfate, 100 mg of Zn as zinc sulfate, 15 mg of Cu as copper sulfate, 0.3 mg of I as ethylene diamine dihydroiodide, and 0.3 mg of Se as sodium selenite.

^3^SID = standardized ileal digestible.

^4^STTD = standardized total tract digestible.

^5^Zinc and formate contents were analyzed by inductive coupled plasma (ICP) spectroscopy and enzymatic assay, respectively (Eurofins Scientific Inc., Des Moines, IA).

**Table 2.** Composition of experimental diets for phase 2

Item	NC	PC	ZnGly 400 mg/kg				ZnGly 800 mg/kg	ZnGly 1,200 mg/kg	ZnO 2,500 mg/kg
			ZnO 0 mg/kg	ZnO 700 mg/kg	ZnO 1,400 mg/kg	ZnO 2,357 mg/kg			
Feedstuff, %									
Corn, yellow	51.44	51.44	51.68	51.61	51.54	51.44	51.64	51.60	51.47
Soybean meal, 48% CP	22.00	22.00	22.00	22.00	22.00	22.00	22.00	22.00	22.00
Whey permeate	13.00	13.00	13.00	13.00	13.00	13.00	13.00	13.00	13.00
Poultry meal	5.00	5.00	5.00	5.00	5.00	5.00	5.00	5.00	5.00
Blood plasma	1.75	1.75	1.75	1.75	1.75	1.75	1.75	1.75	1.75
Fish meal	1.75	1.75	1.75	1.75	1.75	1.75	1.75	1.75	1.75
Poultry fat	2.00	2.00	2.00	2.00	2.00	2.00	2.00	2.00	2.00
Dicalcium phosphate	0.50	0.50	0.50	0.50	0.50	0.50	0.50	0.50	0.50
Limestone	0.75	0.75	0.75	0.75	0.75	0.75	0.75	0.75	0.75
L-Lys HCl	0.50	0.50	0.50	0.50	0.50	0.50	0.50	0.50	0.50
DL-Met	0.20	0.20	0.20	0.20	0.20	0.20	0.20	0.20	0.20
L-Thr	0.16	0.16	0.16	0.16	0.16	0.16	0.16	0.16	0.16
L-Trp	0.01	0.01	0.01	0.01	0.01	0.01	0.01	0.01	0.01
L-Val	0.03	0.03	0.03	0.03	0.03	0.03	0.03	0.03	0.03
Formic acid	0.25	0.25	0.25	0.25	0.25	0.25	0.25	0.25	0.25
Salt	0.22	0.22	0.22	0.22	0.22	0.22	0.22	0.22	0.22
Vitamin premix^1^	0.03	0.03	0.03	0.03	0.03	0.03	0.03	0.03	0.03
Trace mineral premix^2^	0.14	0.14	0.14	0.14	0.14	0.14	0.14	0.14	0.14
Zinc oxide	--	--	--	0.07	0.14	0.24	--	--	0.25
Zinc glycinate	--	--	0.04	0.04	0.04	0.04	0.08	0.12	--
Total	100.00	100.00	100.00	100.00	100.00	100.00	100.00	100.00	100.00
Calculated composition									
DM, %	90.3	90.3	90.2	90.1	90.1	90.0	90.2	90.2	90.1
ME, kcal/kg	3,409	3,409	3,408	3,406	3,403	3,400	3,407	3,405	3,401
CP, %	21.6	21.6	21.6	21.6	21.5	21.5	21.6	21.6	21.5
SID^3^ Lys, %	1.35	1.35	1.35	1.35	1.35	1.35	1.35	1.35	1.35
Ca, %	0.80	0.80	0.80	0.80	0.80	0.80	0.80	0.80	0.80
STTD^4^ P, %	0.40	0.40	0.40	0.40	0.40	0.40	0.40	0.40	0.40
Zn, %	0.02	0.02	0.03	0.07	0.12	0.19	0.04	0.05	0.19
Analyzed composition									
DM, %	90.26	90.17	90.19	90.22	90.53	90.22	89.77	90.33	90.33
CP, %	20.26	20.44	20.83	20.23	21.10	20.02	21.44	21.00	21.42
Zn^5^, %	0.01	0.01	0.02	0.07	0.11	0.18	0.04	0.05	0.18
Formate^5^, %	0.13	0.13	0.15	0.14	0.14	0.14	0.14	0.14	0.14
pH	5.88	5.89	5.75	5.80	5.81	5.78	5.70	5.68	5.85

^1^The vitamin premix provided per kilogram of complete diet: 6,614 IU of vitamin A as vitamin A acetate, 992 IU of vitamin D_3_, 19.8 IU of vitamin E, 2.6 mg of vitamin K as menadione sodium bisulfate, 0.03 mg of vitamin B_12_, 4.6 mg of riboflavin, 18.5 mg of D-pantothenic acid as calcium panthonate, 26.5 mg of niacin, and 0.07 mg of biotin.

^2^The trace mineral premix provided per kilogram of complete diet: 30 mg of Mn as manganous oxide, 100 mg of Fe as ferrous sulfate, 100 mg of Zn as zinc sulfate, 15 mg of Cu as copper sulfate, 0.3 mg of I as ethylene diamine dihydroiodide, and 0.3 mg of Se as sodium selenite.

^3^SID = standardized ileal digestible.

^4^STTD = standardized total tract digestible.

^5^Zinc and formate contents were analyzed by inductive coupled plasma (ICP) spectroscopy and enzymatic assay, respectively (Eurofins Scientific Inc., Des Moines, IA).

### Experimental procedures and sample collection

All pigs were fed the experimental diets for 7 d (pre challenge period) until 64 pigs were orally inoculated with F18^+^*E. coli* on day 7 of the study. The inoculum of F18^+^*E. coli* was prepared to be nalidixic acid resistant and produce heat-stable toxins A (STa) and heat-stable toxins B (STb), using a strain originally resistant to nalidixic acid following the standard protocol as previously described by Cutler et al. (2007). The final concentration of F18^+^*E. coli* was 6 × 10^9^ CFU/mL, and was orally administered in two doses. The unchallenged group (eight pigs) received an oral dose of sterile physiological saline. The fecal score was also recorded during the study to analyze the effects of F18^+^*E. coli* challenge as described in previous studies (Duarte et al., 2020; Xu et al., 2022). The fecal score was recorded using a 1 to 5 scale: 1) very firm stool, 2) normal firm stool, 3) moderately loose stool, 4) loose, watery stool, and 5) very watery stool as described in previous studies (Deng et al., 2022; Xu et al., 2022).

At the end of feeding in the experiment, all pigs were euthanized by exsanguination after the penetration of a captive bolt. After euthanasia, jejunal mucosa and tissues were gently washed with saline to remove the digesta and then collected. Jejunal tissues (15 cm) were obtained and flushed with saline solution to remove digesta. The first 5 cm was stored in 10% formalin buffer for histology and measurement of crypt cell proliferation of jejunal tissue, and the remaining 10 cm was used to collect jejunal mucosa by scraping of the mucosal layer of the jejunum using glass microscope slides. The mucosa samples were frozen in liquid N immediately after sampling and then stored in freezer at −80 °C until further analysis.

### Histology and immunohistochemistry

Two parts of mid-jejunum fixed in 10% buffered formalin for 48 h after tissue sampling, and then transferred to a 70% ethanol solution and sent to the North Carolina State University Histology Laboratory (College of Veterinary Medicine, Raleigh, NC) for dehydration, embedment, and staining according to their internal standard protocol. Villus height and crypt depth were measured using a camera (Infinity 2-2 digital CCD) attached to a microscope (Olympus CX31, Lumenera Corporation, Ottawa, Canada) as described in previous studies (Duarte et al., 2021; Deng et al., 2022). Lengths of 15 well-oriented intact villi and their associated crypts were measured in each slide. The villi length was measured from the top of the villi to the villi–crypt junction, and the crypt depth was measured from the villi–crypt junction to the bottom of the crypt. Then, the villus height to crypt depth (VH:CD) ratio was calculated. Images of 15 intact crypts from each slide were cropped and the Image JS software was used for calculating the ratio of Ki-67 positive cells to total cells in the crypt (%). Morphological evaluation and crypt cell proliferation were executed by the same person.

### Immune status and oxidative stress

Jejunal mucosa was ground using a homogenizer (Tissuemiser, Thermo Fisher Scientific Inc., Rockford, IL, USA) on ice in 2 mL phosphate-buffered saline (PBS) for 30 s. Homogenized samples were centrifuged at 14,000 × *g* for 15 min and then the supernatant was collected. Sample preparation for analysis was followed as described in previous studies (Jang et al., 2020). Protein contents in supernatants were measured using Pierce BCA Protein Assay Kit (23225#, Thermo Fisher Scientific Inc.). Concentrations of tumor necrosis factor-α (TNF-α) and interleukin-8 (IL-8) in the jejunal mucosa samples were determined using ELISA kits (R&D Systems, Minneapolis, MN) following the manufacturer’s protocols. The concentrations of malondialdehyde (MDA) and protein carbonyl were measured by commercial assay kits (Cell Biolabs, Inc., San Diego, CA) following the manufacturers’ protocols. Total concentrations of immunoglobulins (IgG and IgA) in the jejunal mucosa of pigs were measured using an ELISA Kit for swine IgG and IgA (Bethyl Laboratories Inc., Montgomery, TX), respectively, following the manufacturer’s protocols.

### Relative mRNA expression of Zn and amino acid transporters

Frozen jejunal mucosa (50 to 100 mg) was homogenized in 1 mL of trizol reagent, (15-596-026, Invitrogen, Waltham, MA) using a Bead Mill 24 homogenizer (Thermo Fisher), twice at 4.5 m/s for 30 s and rested intermittently for 20 s on ice. Homogenized samples were centrifuged for 10 min at 12,000 × *g* in 4 °C and then the supernatant was transferred to a 1.5 mL centrifuge tube and mixed with 200 μL of chloroform (Thermo Fisher) by gently shaking for 1 min. The tube was then incubated at room temperature for 10 min, and centrifuged 15 min at 12,000 × *g* at 4 °C. The aqueous phase was carefully removed, placed into a new tube, and mixed with 200 μL of isopropanol by gently shaking the tubes for 1 min. Samples were rested for 10 min at room temperature and subsequently centrifuged at 12,000 × *g* for 15 min at 4 °C. All the supernatant was removed and mixed with 75% ethanol. This mix was filtered in columns from the RNeasy Mini kit (Qiagen, Valenica, CA, USA). Columns were washed once with 700 μL of RW1 buffer (catalog no. 1053394, Qiagen) and three times with 500 μL of RPE buffer (catalog no. 1018013, Qiagen). The RNA was eluted with RNase-free water (Invitrogen). The quantity and quality of total RNA were determined using spectrometry. The extracted RNA was reverse transcribed into cDNA using commercial kit (RevertAid First Strand cDNA Synthesis, Thermo Fisher) according to the manufacturer’s instructions. Quantitative real-time polymerase chain reaction (RT–qPCR) was performed using the CFX Connect Real-Time PCR Detection System (BioRad, Hercules, CA, USA) and Maxima SYBR Green/ROX qPCR Master Mix (Thermo Fisher) with oligonucleotide primers synthesized by Millipore Sigma (Burlington, MA). The primers used for target gene are described in Table 3 and β-actin was used as the house-keeping gene. Delta–delta Ct values were calculated to acquire relative mRNA levels per sample. Relative expression was normalized and expressed as a ratio to the expression of targeting genes in the no challenge group.

**Table 3.** Sequence of primers for nutrient transporters in the jejunum of nursery pigs

Item^1^	Forward primers	Reverse primers	Accession number^2^
ZIP 4	CTTCTTCGCCGATGCCAAGG	GTCGCTGAGGTAAAGGGCAG	XM_021090449.1
ZnT1	TCCAACGGGCTGAAATTGGA	ACATCTCCCCCTCAGGACAA	NM_001139470.1
ZnT5	TGCTCGCTATGGATCCGTTC	TAGCGCTCACTACCACTCCT	NM_001001266.2
GLYT1	AGTTCAGAGAGGGGAGGGACTT	AGCACCATTCAGCATCCCTTT	XM_021096812.1
b^0,+^AT	TCTTGCGACACTGGGCTG	ACAGACAGTTTTGTGCCCTCA	EU155139
y+LAT1	TTCCCAAGGTTGCAGCTCAT	TTTGGGGGAGACGAAGATGC	NM_001110421.1
β-Actin	CTCCAGAGCGCAAGTACTCC	GAATGCAACTAACAGTCCGCC	XM_003124280.5

^1^ZIP4, zinc/iron-regulated transporter-like protein 4 (SLC39A4); ZnT1, zinc transporter 1 (SLC30A1); ZnT5, zinc transporter 5 (SLC30A5); GLYT1, glycine transporter 1 (SLC6A9); b^0,+^AT, neutral amino acid transport system b^0,+^ (SLC7A9); y+LAT1, y+L cation and neutral amino acid transporter 1 (SLC7A7).

^2^Primers were designed according to their published sequences at the Genbank database (National Center for Biotechnology Information, Bethesda, MD).

### Microbiome sequencing

The deoxyribonucleic acid (DNA) was extracted from 250 mg of jejunal mucosa samples using a commercial DNA extraction kit (DNA Stool MiniKit, QIAGEN, Germany). Extracted DNA samples were sent to Diversigen, Inc. (Houston, TX) and prepared for shotgun sequencing to estimate the diversity and relative abundance at phylum and family levels of jejunal mucosa-associated microbiota of the pigs. Operational taxonomic units (OTUs) data were transformed to relative abundance (%) for further statistical analysis, and the OTU data with less than 0.05% relative abundance within each level were combined as “other”.

### Statistical analysis

Data were analyzed by the UNIVARIATE procedures (SAS Inst. Inc., Cary, NC) to test for homogeneity, normality, and outliers. The experimental unit was the pen and data were then analyzed by Mixed procedure of SAS 9.4 (SAS Inst. Inc., Cary, NC) as a randomized complete block design. Effects of ZnGly or ZnO levels were analyzed using the polynomial contrast statements to determine linear and quadratic effects with coefficients for equally or unequallyspaced treatments, respectively, by Proc IML procedure of SAS 9.4. Treatment comparisons were made using the following orthogonal contrasts: 1) effect of F18+ *E. coli* challenge (NC vs. PC), 2) effect of pharmaceutical level of ZnO (PC vs. ZnO at 2,500 mg/kg), and 3) effect of zinc source (ZnGly at 400 mg/kg vs. ZnO at 2,500 mg/kg), and effect of zinc supplements [PC vs.(ZnGly at 400 mg/kg + ZnO at 2,500 mg/kg)]. Statistical significance and tendency were considered at *P* < 0.05 and 0.05 ≤ *P* < 0.10, respectively.

## Results

### F18^+^*E. coli* challenge

Initial BW of pigs at the beginning of experiment was 6.5 kg and there was no difference among treatments (Table 4). There was no mortality during overall period in the study. However, three pigs were removed from the study due to severe BW loss (confirmed as outliers): 1) one pig in NC group, 2) one pig in the group fed ZnO at 2,500 mg/kg, and 3) one pig in the group fed ZnGly at 400 mg/kg and ZnO at 700 mg/kg. Pigs had to be removed following the IACUC guideline. In the post challenge period (days 7 to 28), F18^+^*E. coli* challenge tended to decrease (*P* = 0.065) average daily gain (308 to 244 g/d) during days 7 to 14 and tended to decrease (*P* = 0.076) average daily gain (200 to 155 g/d) of nursery pigs during phase 1 (weaning to days 14). The F18^+^*E. coli* challenge decreased (*P* < 0.05) G:F (0.84 to 0.76) during days 21 to 28 and tended to decrease (*P* = 0.055) G:F (0.82 to 0.69) of nursery pigs during phase 1 (weaning to days 14). In the pre challenge period, there was no difference in fecal score of nursery pigs (Table 5). However, the F18^+^*E. coli* challenge increased (*P* < 0.05) fecal score (3.2 to 4.1) of nursery pigs during days 7 to 14, the first week of post challenge period.

**Table 4.** Growth performance of nursery pigs fed diets with varying levels of zinc glycinate (ZnGly) and zinc oxide (ZnO) under F18^+^*E. coli* challenge^1^

Item	NC^2^	PC	ZnGly 400 mg/kg				ZnGly 800 mg/kg	ZnGly 1,200 mg/kg	ZnO 2,500 mg/kg	SEM	*P-*value							
			ZnO 0 mg/kg	ZnO 700 mg/kg	ZnO 1,400 mg/kg	ZnO 2,357 mg/kg					NC vs. PC^3^	PC vs. ZnO 2,500 mg/kg	ZnO Linear^4^	ZnO Quad.^4^	ZnGly Linear^5^	ZnGly Quad.^5^	Zn Source^6^	PC vs. Zn source^7^
BW, kg																		
dayay 0	6.5	6.5	6.5	6.5	6.5	6.5	6.5	6.5	6.6	0.4	0.974	0.922	0.994	0.862	0.975	0.999	0.935	1.000
day 7	7.2	7.0	7.4	7.2	7.0	7.2	7.2	7.1	7.2	0.4	0.520	0.407	0.421	0.244	0.850	0.216	0.509	0.182
day 14	9.3	8.7	9.2	8.5	8.8	9.6	9.0	8.9	9.5	0.5	0.110	0.052	0.176	0.014	0.796	0.243	0.466	0.061
day 21	12.1	12.0	12.6	11.4	11.8	12.7	12.1	11.8	12.6	0.6	0.870	0.360	0.552	0.023	0.585	0.294	0.959	0.267
day 28	17.0	16.8	17.6	16.2	16.6	17.8	16.9	16.3	17.6	0.7	0.807	0.333	0.587	0.019	0.343	0.170	0.930	0.232
ADG, g/d																		
days 0 to 7	92	66	125	99	69	98	96	96	97	26	0.465	0.390	0.370	0.236	0.819	0.171	0.435	0.151
days 7 to 14	308	244	256	168	259	338	260	250	343	31	0.065	0.007	0.002	0.004	0.842	0.645	0.017	0.068
days 14 to 21	394	470	486	415	431	451	441	419	439	35	0.107	0.519	0.607	0.187	0.186	0.571	0.334	0.850
days 21 to 28	736	689	717	691	677	724	690	642	713	31	0.263	0.555	0.882	0.204	0.196	0.185	0.930	0.454
days 7 to 28	471	467	486	425	456	504	464	437	498	22	0.913	0.307	0.277	0.015	0.211	0.263	0.698	0.328
Phase 1^8^	200	155	191	133	164	218	178	167	210	20	0.076	0.038	0.113	0.005	0.765	0.197	0.458	0.043
Phase 2^9^	557	579	601	553	554	587	566	530	576	26	0.536	0.931	0.798	0.109	0.105	0.253	0.488	0.758
Overall	375	367	396	343	359	403	372	349	393	19	0.776	0.323	0.538	0.013	0.328	0.154	0.914	0.219
ADFI, g/d																		
days 0 to 7	150	123	165	153	126	169	146	130	130	22	0.362	0.826	0.971	0.170	0.985	0.168	0.234	0.347
days 7 to 14	344	324	331	259	329	414	311	288	375	28	0.587	0.188	0.006	0.010	0.277	0.570	0.257	0.373
days 14 to 21	579	606	601	523	545	626	558	524	604	43	0.631	0.966	0.497	0.064	0.109	0.727	0.957	0.934
days 21 to 28	874	902	896	858	853	905	920	808	915	36	0.571	0.793	0.803	0.188	0.090	0.114	0.710	0.928
days 7 to 28	579	611	609	547	576	648	596	540	631	29	0.421	0.607	0.174	0.022	0.066	0.310	0.584	0.776
Phase 1	247	224	248	205	228	291	229	209	246	21	0.399	0.446	0.060	0.015	0.473	0.269	0.935	0.935
Phase 2	707	754	749	691	699	766	739	666	759	35	0.335	0.916	0.591	0.074	0.068	0.309	0.826	0.996
Overall	470	489	498	448	463	528	484	438	503	25	0.588	0.691	0.241	0.023	0.111	0.238	0.901	0.692
G:F																		
days 0 to 7	0.48	0.38	0.60	0.61	0.50	0.57	0.60	0.56	0.66	0.13	0.549	0.114	0.750	0.738	0.348	0.283	0.743	0.102
days 7 to 14	0.92	0.77	0.80	0.70	0.76	0.82	0.84	0.88	0.92	0.07	0.142	0.168	0.620	0.318	0.265	0.914	0.244	0.344
days 14 to 21	0.70	0.78	0.81	0.80	0.79	0.73	0.79	0.81	0.73	0.05	0.170	0.362	0.187	0.589	0.790	0.900	0.188	0.767
days 21 to 28	0.84	0.76	0.81	0.81	0.80	0.80	0.75	0.80	0.78	0.03	0.036	0.540	0.808	0.923	0.665	0.977	0.549	0.283
days 7 to 28	0.82	0.77	0.80	0.78	0.79	0.78	0.78	0.81	0.79	0.03	0.222	0.556	0.604	0.867	0.375	0.977	0.760	0.382
Phase 1	0.82	0.69	0.77	0.65	0.69	0.75	0.79	0.81	0.86	0.05	0.055	0.020	0.994	0.095	0.092	0.506	0.208	0.042
Phase 2	0.79	0.77	0.81	0.80	0.80	0.77	0.77	0.80	0.76	0.03	0.575	0.816	0.274	0.749	0.700	0.920	0.205	0.632
Overall	0.80	0.75	0.80	0.77	0.78	0.76	0.77	0.80	0.79	0.03	0.177	0.365	0.378	0.685	0.271	0.772	0.715	0.200

^1^Data represent the least square means of eight replications except for three treatments: 1) one pig in NC group, 2) one pig in the group fed ZnO at 2,500 mg/kg, and 3) one pig in the group fed ZnGly at 400 mg/kg and ZnO at 700 mg/kg. Pigs had to be removed due to severe BW loss following the IACUC guideline after the statistical confirmation as outliers.

^2^No challenge.

^3^F18^+^*E. coli* challenge on d 7 after weaning.

^4^Dose response effects of ZnO supplementation (ZnO at 0, 700, 1,400, and 2,357 mg/kg) with ZnGly at 400 mg/kg.

^5^Dose response effects of ZnGly supplementation [ZnGly at 0 (PC), 400, 800, and 1,200 mg/kg].

^6^ZnGly at 400 mg/kg vs. ZnO at 2,500 mg/kg.

^7^PC vs. ZnGly at 400 mg/kg + ZnO at 2,500 mg/kg.

^8^days 0 to 14.

^9^days 14 to 28.

**Table 5.** Fecal score of nursery pigs fed diets with varying levels of zinc glycinate (ZnGly) and zinc oxide (ZnO) under F18^+^*E. coli* challenge^1^

Item	NC^2^	PC	ZnGly 400 mg/kg				ZnGly 800 mg/kg	ZnGly 1,200 mg/kg	ZnO 2,500 mg/kg	SEM	*P-*value							
			ZnO 0 mg/kg	ZnO 700 mg/kg	ZnO 1,400 mg/kg	ZnO 2,357 mg/kg					NC vs. PC^3^	PC vs. ZnO 2,500 mg/kg	ZnO Linear^4^	ZnO Quad.^4^	ZnGly Linear^5^	ZnGly Quad.^5^	Zn Source^6^	PC vs. Zn source^7^
Fecal score^8^																		
Pre challenge	3.7	3.5	3.6	3.6	3.8	3.5	3.6	3.4	3.1	0.2	0.374	0.122	0.670	0.340	0.693	0.472	0.055	0.498
Post challenge	3.3	3.6	3.6	3.8	3.6	3.5	3.6	3.4	3.4	0.1	0.040	0.211	0.193	0.174	0.242	0.534	0.226	0.454
days 7 to 14	3.2	4.1	4.0	4.4	3.9	3.5	3.8	3.5	3.5	0.2	0.001	0.023	0.024	0.056	0.019	0.673	0.080	0.101
days 14 to 21	3.4	3.4	3.3	3.4	3.4	3.4	3.5	3.5	3.4	0.1	0.705	0.929	0.657	0.841	0.298	0.967	0.684	0.894
days 21 to 28	3.3	3.3	3.5	3.5	3.6	3.5	3.3	3.4	3.4	0.1	0.931	0.439	0.886	0.853	0.957	0.274	0.344	0.152

^1^Data represent the least square means of 8 replications except for three treatments: 1) one pig in NC group, 2) one pig in the group fed ZnO at 2,500 mg/kg, and 3) one pig in the group fed ZnGly at 400 mg/kg and ZnO at 700 mg/kg. Pigs had to be removed due to severe BW loss following the IACUC guideline after the statistical confirmation as outliers.

^2^No challenge.

^3^F18^+^*E. coli* challenge on d 7 after weaning.

^4^Dose response effects of ZnO supplementation (ZnO at 0, 700, 1,400, and 2,357 mg/kg) with ZnGly at 400 mg/kg.

^5^Dose response effects of ZnGly supplementation [ZnGly at 0 (PC), 400, 800, and 1,200 mg/kg].

^6^ZnGly at 400 mg/kg vs. ZnO at 2,500 mg/kg.

^7^PC vs. ZnGly at 400 mg/kg + ZnO at 2,500 mg/kg.

^8^Fecal score is measured from day 2 based on a 1 to 5 scale: 1 (dried feces) to 5 (watery diarrhea).

The F18^+^*E. coli* challenge decreased (*P* < 0.05) villus height to crypt depth ratio (2.00 to 1.70 μm) and increased (*P* < 0.05) enterocyte proliferation (19.1 to 27.5%) in the jejunum of nursery pigs (Table 6). The F18^+^*E. coli* challenge tended to increase TNF-α (*P* = 0.057; 1.31 to 1.87 pg/mg of protein), IgG (*P* = 0.089; 1.12 to 1.80 μg/mg), protein carbonyl (*P* = 0.075; 2.48 to 4.13 nmol/mg), and MDA (*P* = 0.079; 0.31 to 0.44 μM/mg) in the jejunum of nursery pigs (Table 7).

**Table 6.** Jejunal morphology and crypt cell proliferation of nursery pigs fed diets with varying levels of zinc glycinate (ZnGly) and zinc oxide (ZnO) under F18^+^*E. coli* challenge^1^

Item	NC^2^	PC	ZnGly 400 mg/kg				ZnGly 800 mg/kg	ZnGly 1,200 mg/kg	ZnO 2,500 mg/kg	SEM	*P-*value							
			ZnO 0 mg/kg	ZnO 700 mg/kg	ZnO 1,400 mg/kg	ZnO 2,357 mg/kg					NC vs. PC^3^	PC vs. ZnO 2,500 mg/kg	ZnO Linear^4^	ZnO Quad.^4^	ZnGly Linear^5^	ZnGly Quad.^5^	Zn Source^6^	PC vs. Zn source^7^
Jejunum																		
Villus height, μm	516	465	438	504	461	515	494	475	485	30	0.148	0.554	0.092	0.849	0.438	0.869	0.179	0.899
Crypt depth, μm	265	285	245	290	256	285	274	270	265	11	0.123	0.124	0.041	0.475	0.731	0.049	0.114	0.010
VH:CD	2.00	1.70	1.88	1.78	1.86	1.85	1.84	1.80	1.87	0.11	0.046	0.239	0.990	0.711	0.599	0.284	0.942	0.177
Crypt cell proliferation, %	19.1	27.5	25.7	25.6	26.0	26.3	26.5	27.2	26.8	2.3	<0.001	0.727	0.541	0.747	0.912	0.603	0.350	0.358

^1^Data represent the least square means of eight replications except for three treatments: 1) one pig in NC group, 2) one pig in the group fed ZnO at 2,500 mg/kg, and 3) one pig in the group fed ZnGly at 400 mg/kg and ZnO at 700 mg/kg. Pigs had to be removed due to severe BW loss following the IACUC guideline after the statistical confirmation as outliers.

^2^No challenge.

^3^F18^+^*E. coli* challenge on day 7 after weaning.

^4^Dose response effects of ZnO supplementation (ZnO at 0, 700, 1,400, and 2,357 mg/kg) with ZnGly at 400 mg/kg.

^5^Dose response effects of ZnGly supplementation [ZnGly at 0 (PC), 400, 800, and 1,200 mg/kg].

^6^ZnGly at 400 mg/kg vs. ZnO at 2,500 mg/kg.

^7^PC vs. ZnGly at 400 mg/kg + ZnO at 2,500 mg/kg.

**Table 7.** Jejunal immune and oxidative stress response of nursery pigs fed diets with varying levels of zinc glycinate (ZnGly) and zinc oxide (ZnO) under F18^+^*E. coli* challenge^1^

Item	NC^2^	PC	ZnGly at 400 mg/kg				ZnGly 800 mg/kg	ZnGly 1,200 mg/kg	ZnO 2,500 mg/kg	SEM	*P-*value							
			ZnO 0 mg/kg	ZnO 700 mg/kg	ZnO 1,400 mg/kg	ZnO 2,357 mg/kg					NC vs. PC^3^	PC vs. ZnO 2,500 mg/kg	ZnO Linear^4^	ZnO Quad.^4^	ZnGly Linear^5^	ZnGly Quad.^5^	Zn Source^6^	PC vs. Zn source^7^
Jejunal mucosa/mg of protein																		
TNF-α, pg	1.31	1.87	1.33	1.29	1.52	1.32	1.30	1.52	1.01	0.22	0.057	0.004	0.882	0.650	0.236	0.065	0.285	0.006
IL-8, ng	0.71	0.69	0.58	0.69	0.77	0.94	0.54	0.84	0.71	0.12	0.912	0.925	0.034	0.912	0.455	0.093	0.468	0.742
IgG, μg	1.12	1.80	1.66	1.74	1.54	1.80	1.80	2.20	1.78	0.33	0.089	0.947	0.827	0.715	0.282	0.318	0.763	0.798
IgA, μg	6.47	7.67	7.42	8.10	6.13	6.90	6.22	5.87	7.85	1.50	0.549	0.929	0.600	0.880	0.301	0.973	0.828	0.982
Protein carbonyl, nmol	2.48	4.13	2.26	2.55	1.97	2.67	2.37	2.94	2.96	0.89	0.075	0.200	0.779	0.704	0.216	0.054	0.446	0.057
MDA, μM	0.31	0.44	0.34	0.31	0.41	0.49	0.48	0.37	0.43	0.06	0.079	0.842	0.014	0.362	0.711	0.855	0.855	0.390

^1^Data represent the least square means of eight replications except for three treatments: 1) one pig in NC group, 2) one pig in the group fed ZnO at 2,500 mg/kg, and 3) one pig in the group fed ZnGly at 400 mg/kg and ZnO at 700 mg/kg. Pigs had to be removed due to severe BW loss following the IACUC guideline after the statistical confirmation as outliers.

^2^No challenge.

^3^F18^+^*E. coli* challenge on day 7 after weaning.

^4^Dose response effects of ZnO supplementation (ZnO at 0, 700, 1,400, and 2,357 mg/kg) with ZnGly at 400 mg/kg.

^5^Dose response effects of ZnGly supplementation [ZnGly at 0 (PC), 400, 800, and 1,200 mg/kg].

^6^ZnGly at 400 mg/kg vs. ZnO at 2,500 mg/kg.

^7^PC vs. ZnGly at 400 mg/kg + ZnO at 2,500 mg/kg.

Expression of Zn transporters including ZIP4 (*P* = 0.089; 1.04 to 0.69) and ZnT1 (*P* = 0.082; 1.01 to 0.60) tended to be downregulated in the jejunum of nursery pigs challenged with F18^+^*E. coli* compared with no challenge group, whereas ZnT5 expression did not differ in F18^+^*E. coli* challenge group (Table 8). The F18^+^*E. coli* challenge did not affect (*P* > 0.10) expression of the amino acid transporters including GLYT1, b^0,+^AT, and y+LAT1 in the jejunum of nursery pigs. The F18^+^*E. coli* challenge did not affect diversity estimates and relative abundance of bacteria at phylum and family levels in jejunal mucosa of nursery pigs (Tables 9–11).

**Table 10.** Relative abundance of jejunal mucosa-associated microbiota at phylum level of nursery pigs fed diets with varying levels of zinc glycinate (ZnGly) and zinc oxide (ZnO) under F18^+^*E. coli* challenge^1^

Item	NC^2^	PC	ZnGly at 400 mg/kg				ZnGly 800 mg/kg	ZnGly 1,200 mg/kg	ZnO 2,500 mg/kg		*P-*value							
			ZnO 0 mg/kg	ZnO 700 mg/kg	ZnO 1,400 mg/kg	ZnO 2,357 mg/kg				SEM	NC vs. PC^3^	PC vs. ZnO 2,500 mg/kg	ZnO Linear^4^	ZnO Quad.^4^	ZnGly Linear^5^	ZnGly Quad.^5^	Zn Source^6^	PC vs. Zn source^7^
Jejunal mucosa																		
Actinobacteria	13.56	13.10	14.63	12.89	14.12	16.52	15.92	16.26	12.56	1.09	0.754	0.717	0.116	0.075	0.026	0.570	0.167	0.700
Bacteroidetes	3.14	3.19	3.14	3.30	3.21	3.05	3.37	3.21	3.47	0.27	0.901	0.430	0.721	0.532	0.786	0.826	0.352	0.708
Cyanobacteria	0.90	0.98	0.72	0.90	0.72	1.11	0.70	0.95	0.88	0.10	0.565	0.487	0.022	0.299	0.816	0.016	0.265	0.151
Firmicutes	35.79	35.48	31.98	35.92	32.66	29.74	29.14	32.03	37.06	1.95	0.913	0.569	0.219	0.120	0.135	0.107	0.071	0.688
Proteobacteria	40.28	40.42	43.77	41.35	43.49	43.59	45.51	41.81	39.64	1.70	0.953	0.746	0.816	0.525	0.440	0.043	0.091	0.539
Spirochaetes	1.98	2.33	1.90	1.62	1.88	2.13	1.71	2.14	2.00	0.29	0.395	0.422	0.441	0.418	0.554	0.147	0.816	0.290
Others	4.36	4.49	3.84	4.00	3.90	3.84	3.63	3.60	4.38	0.26	0.719	0.768	0.924	0.696	0.013	0.225	0.133	0.227
F:B^8^	11.50	11.62	10.48	10.98	10.50	10.46	9.08	10.78	10.99	1.04	0.932	0.664	0.898	0.819	0.397	0.172	0.726	0.482

^1^Data represent the least square means of 8 replications except for 3 treatments: (1) one pig in NC group, (2) one pig in the group fed ZnO at 2,500 mg/kg, and (3) one pig in the group fed ZnGly at 400 mg/kg and ZnO at 700 mg/kg. Pigs had to be removed due to severe BW loss following the IACUC guideline after the statistical confirmation as outliers.

^2^No challenge.

^3^F18^+^*E. coli* challenge on day 7 after weaning.

^4^Dose response effects of ZnO supplementation (ZnO at 0, 700, 1,400, and 2,357 mg/kg) with ZnGly at 400 mg/kg.

^5^Dose response effects of ZnGly supplementation [ZnGly at 0 (PC), 400, 800, and 1,200 mg/kg].

^6^ZnGly at 400 mg/kg vs. ZnO at 2,500 mg/kg.

^7^PC vs. ZnGly at 400 mg/kg + ZnO at 2,500 mg/kg.

^8^Firmicutes to Bacteroidetes ratio.

**Table 11.** Relative abundance of jejunal mucosa-associated microbiota at family level of nursery pigs fed diets with varying levels of zinc glycinate (ZnGly) and zinc oxide (ZnO) under F18^+^*E. coli* challenge^1^

Item	NC^2^	PC	ZnGly at 400 mg/kg				ZnGly 800 mg/kg	ZnGly 1,200 mg/kg	ZnO 2,500 mg/kg		*P-*value							
			ZnO 0 mg/kg	ZnO 700 mg/kg	ZnO 1,400 mg/kg	ZnO 2,357 mg/kg				SEM	NC vs. PC^3^	PC vs. ZnO 2,500 mg/kg	ZnO Linear^4^	ZnO Quad.^4^	ZnGly Linear^5^	ZnGly Quad.^5^	Zn Source^6^	PC vs. Zn source^7^
Jejunal mucosa																		
* Alcaligenaceae*	2.19	1.83	2.07	2.10	1.97	2.49	2.40	2.97	2.04	0.29	0.241	0.475	0.201	0.258	0.001	0.441	0.933	0.383
* Bacillaceae*	2.42	2.36	2.12	2.63	2.22	2.05	2.17	2.28	2.57	0.25	0.834	0.506	0.502	0.181	0.841	0.438	0.159	0.958
* Burkholderiaceae*	1.56	1.57	1.27	1.41	1.47	1.36	1.45	1.38	1.27	0.17	0.964	0.210	0.702	0.445	0.612	0.497	0.995	0.150
* Clostridiaceae*	16.10	16.22	13.84	17.34	13.56	12.69	12.15	13.47	16.48	1.31	0.951	0.888	0.203	0.170	0.097	0.165	0.161	0.514
* Comamonadaceae*	2.33	3.14	4.85	3.19	3.60	4.21	3.39	3.76	3.51	0.72	0.382	0.684	0.685	0.090	0.891	0.304	0.150	0.193
* Deinococcaceae*	2.06	1.86	1.59	1.91	1.62	1.73	1.72	1.68	1.83	0.19	0.423	0.891	0.855	0.636	0.606	0.503	0.334	0.474
* Enterobacteriaceae*	6.45	7.02	5.19	4.85	6.36	5.17	5.19	6.80	6.10	0.64	0.482	0.254	0.648	0.334	0.796	0.004	0.257	0.051
* Flavobacteriaceae*	0.97	1.14	1.01	1.14	1.06	1.09	1.61	1.23	1.16	0.16	0.432	0.920	0.823	0.790	0.221	0.417	0.488	0.776
* Lactobacillaceae*	0.47	1.33	0.85	0.42	1.13	0.29	0.52	1.14	0.71	0.50	0.228	0.385	0.619	0.626	0.684	0.276	0.845	0.373
* Methylococcaceae*	0.67	0.77	1.65	0.94	1.07	1.22	1.33	0.57	1.20	0.37	0.820	0.363	0.476	0.200	0.528	0.016	0.331	0.110
* Micromonosporaceae*	1.42	1.36	1.18	1.77	1.80	1.49	2.03	1.99	1.66	0.29	0.841	0.441	0.530	0.126	0.037	0.846	0.251	0.822
* Mycobacteriaceae*	2.26	2.02	1.75	2.01	1.88	1.52	1.86	2.05	1.83	0.20	0.417	0.500	0.328	0.161	0.822	0.257	0.795	0.355
* Nocardiaceae*	0.86	1.35	1.37	1.20	1.11	1.37	1.38	2.05	1.29	0.24	0.132	0.834	0.991	0.339	0.047	0.158	0.797	0.926
* Pseudoalteromonadaceae*	2.87	3.40	2.79	3.20	3.16	2.68	3.00	2.69	3.25	0.23	0.118	0.666	0.609	0.067	0.071	0.535	0.166	0.195
* Pseudomonadaceae*	3.45	3.38	3.76	3.53	3.35	4.17	3.43	3.53	3.41	0.28	0.861	0.955	0.292	0.048	0.931	0.590	0.326	0.527
* Rhizobiaceae*	3.71	2.51	2.71	4.63	2.84	2.37	2.96	2.44	1.83	0.91	0.334	0.583	0.439	0.239	0.993	0.679	0.478	0.823
* Sphingomonadaceae*	5.59	6.77	9.26	5.46	8.54	7.54	8.91	6.80	4.90	0.92	0.362	0.152	0.634	0.198	0.946	0.015	0.001	0.787
* Staphylococcaceae*	13.08	12.12	11.51	11.18	11.96	11.24	10.57	11.57	12.29	1.16	0.529	0.911	0.972	0.820	0.594	0.461	0.612	0.870
* Streptomycetaceae*	6.25	5.52	6.56	6.00	6.69	9.41	7.18	7.21	5.60	0.66	0.437	0.935	0.002	0.028	0.059	0.447	0.306	0.492
* Xanthomonadaceae*	1.79	2.30	1.83	1.79	2.01	2.57	4.38	1.98	2.00	0.64	0.562	0.731	0.359	0.674	0.565	0.124	0.849	0.613

^1^Data represent the least square means of 8eight replications except for three treatments: (1) one pig in NC group, (2) one pig in the group fed ZnO at 2,500 mg/kg, and (3) one pig in the group fed ZnGly at 400 mg/kg and ZnO at 700 mg/kg. Pigs had to be removed due to severe BW loss following the IACUC guideline after the statistical confirmation as outliers.

^2^No challenge.

^3^F18^+^*E. coli* challenge on d 7 after weaning.

^4^Dose–response effects of ZnO supplementation (ZnO at 0, 700, 1,400, and 2,357 mg/kg) with ZnGly at 400 mg/kg.

^5^Dose–response effects of ZnGly supplementation [ZnGly at 0 (PC), 400, 800, and 1,200 mg/kg].

^6^ZnGly at 400 mg/kg vs. ZnO at 2,500 mg/kg.

^7^PC vs. ZnGly at 400 mg/kg + ZnO at 2,500 mg/kg.

**Table 8.** Relative gene expression of zinc and amino acid transporters in the jejunum of nursery pigs fed diets with varying levels of zinc glycinate (ZnGly) and zinc oxide (ZnO) under F18^+^*E. coli* challenge^1^

Item^2^	NC^3^	PC	ZnGly at 400 mg/kg				ZnGly 800 mg/kg	ZnGly 1,200 mg/kg	ZnO 2,500 mg/kg		*P-*value							
			ZnO 0 mg/kg	ZnO 700 mg/kg	ZnO 1,400 mg/kg	ZnO 2,357 mg/kg				SEM	NC vs. PC^4^	PC vs. ZnO 2,500 mg/kg	ZnO Linear^5^	ZnO Quad.^5^	ZnGly Linear^6^	ZnGly Quad.^6^	Zn Source^7^	PC vs. Zn source^8^
Jejunum																		
ZIP4	1.04	0.69	0.89	0.81	0.86	0.77	1.14	1.03	0.63	0.16	0.089	0.772	0.617	0.979	0.057	0.283	0.192	0.697
ZnT1	1.01	0.60	0.69	0.85	0.70	0.75	0.80	0.86	0.65	0.18	0.082	0.826	0.969	0.803	0.235	0.907	0.862	0.723
ZnT5	1.04	0.92	1.13	1.13	0.97	1.10	1.11	1.22	0.89	0.13	0.415	0.824	0.647	0.428	0.075	0.626	0.113	0.506
GLYT1	1.03	0.96	0.79	1.01	0.84	0.88	0.98	1.11	0.87	0.15	0.737	0.661	0.891	0.602	0.343	0.308	0.702	0.469
b^0,+^AT	1.02	0.98	1.06	0.91	0.63	1.04	0.85	1.18	1.06	0.24	0.898	0.817	0.837	0.228	0.698	0.593	0.997	0.788
y+LAT1	1.00	0.93	0.99	0.99	0.93	1.27	1.12	1.16	0.87	0.18	0.696	0.777	0.180	0.232	0.148	0.939	0.579	0.987

^1^Data represent the least square means of eight replications except for three treatments: 1) one pig in NC group, 2) one pig in the group fed ZnO at 2,500 mg/kg, and 3) one pig in the group fed ZnGly at 400 mg/kg and ZnO at 700 mg/kg. Pigs had to be removed due to severe BW loss following the IACUC guideline after the statistical confirmation as outliers.

^2^ZIP4, zinc/iron-regulated transporter-like protein 4 (SLC39A4); ZnT1, zinc transporter 1 (SLC30A1); ZnT5, zinc transporter 5 (SLC30A5); GLYT1, glycine transporter 1 (SLC6A9); b^0,+^AT, neutral amino acid transport system b^0,+^ (SLC7A9); y+LAT1, y+L cation and neutral amino acid transporter 1 (SLC7A7).

^3^No challenge.

^4^F18^+^*E. coli* challenge on day 7 after weaning.

^5^Dose response effects of ZnO supplementation (ZnO at 0, 700, 1,400, and 2,357 mg/kg) with ZnGly at 400 mg/kg.

^6^Dose response effects of ZnGly supplementation [ZnGly at 0 (PC), 400, 800, and 1,200 mg/kg].

^7^ZnGly at 400 mg/kg vs. ZnO at 2,500 mg/kg.

^8^PC vs. ZnGly at 400 mg/kg + ZnO at 2,500 mg/kg.

**Table 9.** Alpha-diversity in jejunal mucosa-associated microbiota of nursery pigs fed diets with varying levels of zinc glycinate (ZnGly) and zinc oxide (ZnO) under F18^+^*E. coli* challenge^1^

Item	NC^2^	PC	ZnGly at 400 mg/kg				ZnGly 800 mg/kg	ZnGly 1,200 mg/kg	ZnO 2,500 mg/kg		*P-*value							
			ZnO 0 mg/kg	ZnO 700 mg/kg	ZnO 1,400 mg/kg	ZnO 2,357 mg/kg				SEM	NC vs. PC^3^	PC vs. ZnO 2,500 mg/kg	ZnO Linear^4^	ZnO Quad.^4^	ZnGly Linear^5^	ZnGly Quad.^5^	Zn Source^6^	PC vs. Zn source^7^
Jejunal mucosa																		
Chao1	146.7	150.6	148.7	146.4	147.8	142.4	149.4	144.6	145.7	2.4	0.182	0.096	0.048	0.473	0.065	0.472	0.301	0.181
Shannon	3.96	4.02	3.99	3.99	4.02	4.08	4.06	4.03	4.02	0.04	0.211	0.956	0.039	0.503	0.480	0.966	0.470	0.724
Simpson	0.947	0.952	0.955	0.951	0.958	0.962	0.961	0.958	0.953	0.003	0.328	0.869	0.070	0.364	0.108	0.374	0.629	0.639

^1^Data represent the least square means of eight replications except for three treatments: 1) one pig in NC group, 2) one pig in the group fed ZnO at 2,500 mg/kg, and 3) one pig in the group fed ZnGly at 400 mg/kg and ZnO at 700 mg/kg. Pigs had to be removed due to severe BW loss following the IACUC guideline after the statistical confirmation as outliers.

^2^No challenge.

^3^F18^+^*E. coli* challenge on d 7 after weaning.

^4^Dose response effects of ZnO supplementation (ZnO at 0, 700, 1,400, and 2,357 mg/kg) with ZnGly at 400 mg/kg.

^5^Dose response effects of ZnGly supplementation [ZnGly at 0 (PC), 400, 800, and 1,200 mg/kg].

^6^ZnGly at 400 mg/kg vs. ZnO at 2,500 mg/kg.

^7^PC vs. ZnGly at 400 mg/kg + ZnO at 2,500 mg/kg.

### The pharmaceutical use of ZnO at 2,500 mg/kg

In the pre challenge period, supplementation of ZnO at 2,500 mg/kg in the diets did not affect growth performance of nursery pigs (Table 4). In the post challenge period, supplementation of ZnO at 2,500 mg/kg in the diets tended to increase (*P* = 0.052) BW (8.7–9.5 kg) of nursery pigs challenged with F18^+^*E. coli* on day 14. Supplementation of ZnO at 2,500 mg/kg increased (*P* < 0.05) ADG during days 7 to 14 (244 to 343 g/d) and phase 1 (155 to 210 g/d) and G:F (0.69–0.86) of nursery pigs challenged with F18^+^*E. coli* during phase 1. Supplementation of ZnO at 2,500 mg/kg decreased (*P <* 0.05) fecal score (4.1–3.5) of nursery pigs challenged with F18^+^*E. coli* during days 7 to 14 (Table 5).

Supplementation of ZnO at 2,500 mg/kg in the diets did not affect the jejunal morphology and enterocyte proliferation of nursery pigs challenged with F18^+^*E. coli* (Table 6). However, supplementation of ZnO at 2,500 mg/kg in the diets decreased (*P* < 0.05) TNF-α (1.87–1.01 pg/mg) in the jejunum of nursery pigs challenged with F18^+^*E. coli* (Table 7). Supplementation of ZnO at 2,500 mg/kg in the diets did not affect gene expressions of zinc and amino acid transporters in the jejunum of nursery pigs challenged with F18^+^*E. coli* (Table 8). Supplementation of ZnO at 2,500 mg/kg in the diets tended to reduce (*P* = 0.096) Chao1 index (150.6–145.7) in the jejunum of nursery pigs challenged with F18^+^*E. coli* (Table 9). However, supplementation of ZnO at 2,500 mg/kg in the diets did not affect the relative abundance of jejunal mucosa-associated microbiota at phylum and family levels of nursery pigs challenged with F18^+^*E. coli* (Tables 10 and 11).

### Dose response of ZnGly on growth and intestinal health of nursery pigs challenged with F18^+^*E. coli*

In the pre challenge period, increasing supplemental levels of ZnGly from 0 to 1,200 mg/kg in the diets did not affect growth performance of nursery pigs (Table 4). In the post challenge period, increasing supplemental levels of ZnGly from 0 to 1,200 mg/kg in the diets tended to linearly decrease average daily feed intake of nursery pigs challenged with F18^+^*E. coli* by 10% (*P* = 0.090; 902 to 808 g/d) during days 21 to 28, by 13% (*P* = 0.066; 611 to 540 g/d) during days 7 to 28, and by 13% (*P* = 0.068; 754 to 666 g/d) during phase 2. Increasing supplemental levels of ZnGly from 0 to 1,200 mg/kg in the diets tended to linearly increase (*P =* 0.092) G:F (0.69–0.81) of nursery pigs challenged with F18^+^*E. coli* by 17% during phase 1.

Increasing supplemental levels of ZnGly from 0 to 1,200 mg/kg in the diets linearly decreased (*P <* 0.05) fecal score (4.1–3.5) of nursery pigs challenged with F18^+^*E. coli* by 15% during days 7–14 (Table 5). Increasing supplemental levels of ZnGly from 0 to 1,200 mg/kg in the diets had quadratic effects (*P <* 0.05) on crypt depth (minimum 259 μm at 654 mg/kg of ZnGly) of nursery pigs challenged with F18^+^*E. coli* (Table 6). Increasing supplemental levels of ZnGly from 0 to 1,200 mg/kg in the diets tended to have quadratic effects on TNF-α (*P =* 0.065; minimum 1.51 pg/mg at 405 mg/kg of ZnGly), IL-8 (*P =* 0.093; minimum 0.53 ng/mg at 494 mg/kg of ZnGly), and protein carbonyl (*P =* 0.054; minimum 2.30 pg/mg at 675 mg/kg of ZnGly) in the jejunum of nursery pigs challenged with F18^+^*E. coli* (Table 7).

Increasing supplemental levels of ZnGly from 0 to 1,200 mg/kg in the diets tended to linearly enhance the expression of Zn transporters including ZIP4 (*P =* 0.057; 0.69 to 1.03) and ZnT5 (*P =* 0.075; 0.92 to 1.22) in the jejunum of nursery pigs challenged with F18^+^*E. coli* (Table 8). However, there was no difference in the expression of the amino acid transporters in jejunum of nursery pigs challenged with F18^+^*E. coli* by increasing supplemental levels of ZnGly from 0 to 1,200 mg/kg in the diets.

Increasing supplemental levels of ZnGly from 0 to 1,200 mg/kg in the diets tended to linearly decrease (*P =* 0.065) Chao1 index (150.6–144.6) in the jejunum of nursery pigs challenged with F18^+^*E. coli* (Table 9). Increasing supplemental levels of ZnGly from 0 to 1,200 mg/kg in the diets linearly increased (*P <* 0.05) relative abundance of Actinobacteria (13.10–16.26%) and decreased (*P <* 0.05) relative abundance of others (4.49–3.60%) at the phylum level in the jejunum of nursery pigs challenged with F18^+^*E. coli* (Table 10). Increasing supplemental levels of ZnGly from 0 to 1,200 mg/kg in the diets had quadratic effects (*P* < 0.05) on relative abundance of Cyanobacteria (minimum 0.67% at 625 mg/kg of ZnGly) and Proteobacteria (minimum 45.6% at 735 mg/kg of ZnGly) at the phylum level in the jejunum of nursery pigs challenged with F18^+^*E. coli*.

Increasing supplemental levels of ZnGly from 0 to 1,200 mg/kg in the diets linearly increased (*P <* 0.05) relative abundance of *Alcaligenaceae* (1.83–2.97%), *Micromonosporaceae* (1.36–1.99%), and *Nocardiaceae* (1.35–2.05%) and tended to linearly decrease relative abundance of *Pseudoalteromonadaceae* (*P =* 0.071; 3.40–2.69%) and increase relative abundance of *Clostridiaceae* (*P =* 0.097; 16.22–13.47%) and *Streptomycetaceae* (*P =* 0.059; 5.52–7.21%) at family level in the jejunum of nursery pigs challenged with F18^+^*E. coli* (Table 11). Increasing supplemental levels of ZnGly from 0 to 1,200 mg/kg in the diets had quadratic effects (*P <* 0.05) on relative abundance of *Enterobacteriaceae* (minimum 4.79% at 660 mg/kg of ZnGly), *Methylococcaceae* (maximum 1.45% at 467 mg/kg of ZnGly), and *Sphingomonadaceae* (maximum 9.40% at 607 mg/kg of ZnGly).

### Dose response of ZnO with ZnGly supplementation at 400 mg/kg on growth and intestinal health of nursery pigs challenged with F18^+^*E. coli*

In the pre challenge period (weaning to day 7), supplementation of ZnGly reducing the levels of ZnO from 2,357 to 0 mg/kg did not affect (*P* > 0.10) the growth performance of nursery pigs (Table 4). In the post challenge period, supplementation of ZnGly reducing the levels of ZnO from 2,357 to 0 mg/kg had quadratic effects (*P* < 0.05) on BW of nursery pigs challenged with F18^+^*E. coli* on day 14 (minimum 8.6 kg at 1,100 mg/kg of ZnO in the diets), day 21 (minimum 11.6 kg at 1,063 mg/kg of ZnO), and day 28 (minimum 16.3 kg at 1,100 mg/kg of ZnO). Supplementation of ZnGly reducing the levels of ZnO from 2,357 to 0 mg/kg linearly decreased (*P* < 0.05) ADG (338 to 256 g/d) of nursery pigs challenged with F18^+^*E. coli* by 24% during days 7 to 14. Supplementation of ZnGly reducing the levels of ZnO from 2,357 to 0 mg/kg had quadratic effects (*P* < 0.05) on ADG of nursery pigs challenged with F18^+^*E. coli* during days 7 to 14 (minimum 211 g/d at 738 mg/kg of ZnO), during days 7 to 28 (minimum 438 g/d at 1,043 mg/kg of ZnO), phase 1 (minimum 145 g/d at 1,013 mg/kg of ZnO), and throughout the overall period (minimum 352 g/d at 1,013 mg/kg of ZnO). Supplementation of ZnGly reducing the levels of ZnO from 2,357 to 0 mg/kg linearly decreased (*P* < 0.05) ADFI (414 to 331 g/d) of nursery pigs challenged with F18^+^*E. coli* by 20% during days 7 to 14 and tended to linearly decrease (*P =* 0.060) ADFI (291 to 248 g/d) of nursery pigs challenged with F18^+^*E. coli* by 15% during phase 1. Supplementation of ZnGly reducing the levels of ZnO from 2,357 to 0 mg/kg had quadratic effects (*P* < 0.05) on ADFI of nursery pigs challenged with F18^+^*E. coli* during days 7 to 14 (minimum 352 g/d at 1,013 mg/kg of ZnO), days 7 to 28 (minimum 556 g/d at 989 mg/kg of ZnO), phase 1 (minimum 213 g/d at 905 mg/kg of ZnO), and throughout the overall period (minimum 447 g/d at 1,105 mg/kg of ZnO). Supplementation of ZnGly reducing the levels of ZnO from 2,357 to 0 mg/kg tended to have quadratic effects on ADFI during days 14 to 21 (*P =* 0.064; minimum 528 g/d at 1,067 mg/kg of ZnO) and phase 2 (*P =* 0.076; minimum 692 g/d at 1,045 mg/kg of ZnO). Supplementation of ZnGly reducing the levels of ZnO from 2,357 to 0 mg/kg tended to have quadratic effects on G:F during phase 1 (*P* = 0.095; minimum 0.68 at 1,000 mg/kg of ZnO).

Supplementation of ZnGly reducing the levels of ZnO from 2,357 to 0 mg/kg linearly increased (*P <* 0.05) fecal score (3.5–4.0) by 14% and tended to have quadratic response on the fecal score (maximum 4.2 at 667 mg/kg of ZnO) of nursery pigs challenged with F18^+^*E. coli* during days 7 to 14 during post challenge period (Table 5). Supplementation of ZnGly reducing the levels of ZnO from 2,357 to 0 mg/kg linearly decreased (*P <* 0.05) crypt depth (285–245 μm) by 16% and enterocyte proliferation (26.3–25.7%) by 2.3% and tended to linearly decrease villus height (515–438 μm) by 18% in the jejunum of nursery pigs challenged with F18^+^*E. coli* (Table 6). Supplementation of ZnGly reducing the levels of ZnO from 2,357 to 0 mg/kg linearly decreased (*P <* 0.05) IL-8 (0.94–0.58 ng/mg of protein) by 39% and MDA (0.49–0.34 μM/mg of protein) by 31% in jejunum of nursery pigs challenged with F18^+^*E. coli* (Table 7). However, supplementation of ZnGly reducing the levels of ZnO from 2,357 to 0 mg/kg did not affect the expression of Zn and amino acid transporters in the jejunum of nursery pigs challenged with F18^+^*E. coli* (Table 8).

Supplementation of ZnGly reducing the levels of ZnO from 2,357 to 0 mg/kg linearly increased (*P* < 0.05) Chao1 index (142.2–148.7) by 5% and decreased (*P* < 0.05) Shannon index (4.08–3.99) by 2% and tended to linearly decrease (*P =* 0.070) Simpson index (0.962–0.955) by 0.7% in jejunum of nursery pigs challenged with F18^+^*E. coli* (Table 9). Supplementation of ZnGly reducing the levels of ZnO from 2,357 to 0 mg/kg linearly decreased (*P <* 0.05) relative abundance of Actinobacteria (16.52–14.63%) by 11% at the phylum level in the jejunum of nursery pigs challenged with F18^+^*E. coli* (Table 10). Supplementation of ZnGly reducing the levels of ZnO from 2,357 to 0 mg/kg tended to have quadratic effects (*P* = 0.075) on relative abundance of Actinobacteria at the phylum level (minimum 13.59% at 675 mg/kg of ZnO) in the jejunum of nursery pigs challenged with F18^+^*E. coli*. Supplementation of ZnGly reducing the levels of ZnO from 2,357 to 0 mg/kg linearly decreased (*P <* 0.05) relative abundance of *Streptomycetaceae* (9.41–6.56%) by 30% at family level in the jejunum of nursery pigs challenged with F18^+^*E. coli* (Table 11). Supplementation of ZnGly reducing the levels of ZnO from 2,357 to 0 mg/kg had quadratic effects (*P* < 0.05) on relative abundance of *Pseudomonadaceae* (minimum 6.15% at 1,000 mg/kg of ZnO) and *Streptomycetaceae* (minimum 5.91% at 800 mg/kg of ZnO) at the family level in the jejunum of nursery pigs challenged with F18^+^*E. coli*. Supplementation of ZnGly reducing the levels of ZnO from 2,357 to 0 mg/kg tended to have quadratic effects on relative abundance of *Comamonadaceae* (*P* = 0.090; minimum 3.29% at 1,278 mg/kg of ZnO) and *Pseudoalteromonadaceae* (*P* = 0.067; maximum 3.03% at 571 mg/kg of ZnO) at the family level in the jejunum of nursery pigs challenged with F18^+^*E. coli*.

## Discussion

Enterotoxigenic *E. coli* is one of the most prominent bacteria responsible for post weaning diarrhea, which negatively impacts swine production. Previous studies demonstrated that enterotoxigenic *E. coli* attaches to glycoprotein receptors on enterocytes, produces enterotoxins including STa and STb, and induces the imbalance of microbiota in the intestine (Fairbrother et al., 2005; Duarte and Kim, 2022), leading to immune system activation, diarrhea, and thus, growth retardation (Sun and Kim, 2017; Duarte et al., 2020; Sun et al., 2021). In particular, nursery pigs are susceptible to the negative impacts of enterotoxigenic *E. coli* due to their immature intestinal development (Moeser et al., 2007; Sun and Kim, 2017). Previous studies also showed that pigs challenged with enterotoxigenic *E. coli* had impaired intestinal barrier functions, and increasing inflammation and oxidative stress with severe diarrhea (Duarte et al., 2020; Xu et al., 2022). In this study, the F18^+^*E. coli* challenge successfully impaired the intestinal health of nursery pigs by increasing inflammation and oxidative stress in the jejunal epithelium resulting in damages to intestinal villi and in turn increasing enterocyte proliferation. However, the growth, fecal score, and some intestinal health parameters partly recovered during the second phase of this study. Although the fecal score was increased by the F18^+^*E. coli* challenge right after inoculation, the extent of increase was smaller than the results in previous reports (Duarte et al., 2020; Sun et al., 2021).

ZnO has been one of the most common feed additives used in swine diets to control enteric disease and promote the growth of pigs (Poulsen, 1995). However, most countries have legally reduced the excessive use of ZnO in the feeds due to health and environmental concerns (Szuba-Trznadel et al., 2021). With the increase in the need to find alternatives to reducing the pharmaceutical use of ZnO, ZnGly, as an organic form of Zn source in the feeds, has been suggested to replace the use of inorganic Zn sources for the pigs through the relatively high utilization of Zn in the body (Diao et al., 2021). The results of this study show that ZnGly supplementation, especially at a range of 400 to 675 mg/kg in nursery feeds, could reduce the negative impacts of F18^+^*E. coli* on the growth and intestinal health of nursery pigs. In addition, ZnGly supplementation at 400 mg/kg in nursery feeds have similar effects to the pharmaceutical use of ZnO, without negatively affecting growth performance of the pigs. Therefore, this study indicates that ZnGly supplementation could be an alternative to the pharmaceutical use of ZnO in the nursery feeds for growth and intestinal health of pigs under the F18^+^*E. coli* challenge, reducing Zn supplementation from 1,813 to 104 mg/kg.

Zn is an essential micro-mineral involved in various physiological responses related to cell repair and division, and immune and oxidative responses, possibly due to its function as a cofactor for over 300 metalloenzymes in the body (McCall et al., 2000). In this study, the effects of ZnGly supplementation at a range of 405 to 675 mg/kg in the feeds considerably reduced the concentrations of proinflammatory cytokines, including TNF-α and IL-8, and protein carbonyl, in addition to decreased crypt depth in the jejunum of nursery pigs challenged with F18^+^*E. coli*. These results could be related to the need for increased Zn intake, which is significantly elevated by enterotoxigenic *E. coli* challenge during the postweaning period (Maywald et al., 2017). Mocchegiani and Malavolta (2007) also showed that proinflammatory cytokines could promote the expression of metallothionein and alpha-2-macroglobulin, which are involved in cellular Zn homeostasis and responding to stress and inflammation. According to the NRC (2012), growing pigs need Zn at 100 mg/kg in the feeds for growth and maintenance. In this study, Zn was supplied as Zn sulfate from a mineral premix in the diets to meet the Zn requirement. In this study, the pigs, were not fed with additional Zn sources, were more susceptible to adverse impacts of F18+ *E. coli* on intestinal health through activation of the immune and oxidative stress responses in the jejunum. Maywald et al. (2017) also described that Zn deficiency could increase susceptibility to inflammatory responses due to acute disruption of the activities and differentiation of immune cells. Kloubert et al. (2018) reported that the Zn-deficient pigs had impaired innate and adaptive immunity with decreased phagocytosis and imbalanced differentiation of T-lymphocytes. Zn can also be essential in alleviating oxidative stress by improving total antioxidant capacity and decreasing activity of superoxide dismutase (Hao et al., 2021).

This study also suggests that the positive changes in intestinal immune and oxidative stress responses of the pigs could be related to glycine as an amino acid compound in ZnGly. According to Yang and Liao (2019), glycine can effectively protect enterocytes from harmful agents because glycine can be a substrate for synthesizing glutathione as an agent to protect from oxidative damage and can also be conjugated with toxin compounds for biochemical detoxification. Ji et al. (2022) showed that glycine supplementation could positively regulate mucosal immunity through the deactivation of gene expression involved in the secretion and signaling of proinflammatory cytokines in the jejunum of nursery pigs. Meléndez-Hevia et al. (2021) also described that glycine could be utilized to synthesize collagen as the main protein of the extracellular matrix, physically protecting the cells from the invasions of microorganisms. According to previous study, supplementation of ZnGly could improve intestinal barrier function and increase gene expression of IL-1β in the jejunal mucosa of nursery pigs (Diao et al., 2021). Therefore, ZnGly supplementation, especially a range of 405 to 675 mg/kg in the feeds, may help to support Zn homeostasis under F18^+^*E. coli* challenge to alleviate inflammation and oxidative stress in the jejunum of nursery pigs.

Dietary Zn absorption can be related to a regulatory mechanism to mediate intestinal expression of Zn transporters. In this study, RT–PCR was performed to accurately determine the jejunal mRNA abundance of Zn and amino acid transporters to investigate how ZnGly supplementation can improve the Zn absorption in the jejunum of nursery pigs challenged with F18^+^*E. coli*. The ZIP4, ZnT1, and ZnT5 are the main Zn transporters, and GLYT1, a sodium and chloride–dependent glycine-specific transporter, b0,+AT, a neutral amino acid transporter, and y+LAT1, the alternative light subunit that makes up the heterodimeric transport system y + L for cationic and neutral amino acids. In this study, the F18^+^*E. coli* challenge decreased the mRNA expression of ZIP4 and ZnT1 in the jejunum of nursery pigs. The ZIP4 is a protein predominantly expressed in the small intestine and responsible for Zn uptake from the extracellular space into the cytoplasm, whereas ZnT1 is a protein of a major Zn transporter abundant along the basolateral membranes of enterocytes and is responsible for cellular Zn efflux to plasma (Cousins, 2010; Hara et al., 2017). Lang et al. (2007) also showed that the inflammation could selectively regulate the mRNA expression of Zn transporters belonging to the Solute Carrier Family 39 (SLC39; ZIP) and Solute Carrier Family 30 (SLC30; ZnT) in a mouse model. Thus, this study shows that the F18^+^*E. coli* challenge may negatively affect the Zn homeostasis in the jejunum of nursery pigs.

Increasing supplemental levels of ZnGly from 0 to 1,200 mg/kg linearly increased the mRNA expression of jejunal ZIP4 and ZnT5 in pigs challenged with F18^+^*E. coli* without affecting mRNA expression of amino acid transporters. The ZnT5 is responsible for Zn transport from the cytoplasm into the Golgi apparatus for secretary granules, and can contribute to the activity of metalloenzymes in the enterocyte (Wang and Zhou, 2010). Interestingly, a previous study showed the opposite result, as supplementation of ZnGly decreased the mRNA expression level of jejunal ZIP4 in nursery pigs, especially under normal conditions (Diao et al., 2021). Thus, the results in this study could indicate that ZnGly would be efficiently utilized to support intestinal Zn homeostasis in the intestine of nursery pigs under stressful or disease conditions. Furthermore, supplementation of ZnO at various levels did not change the mRNA expression of Zn and amino acid transporters in the jejunum of nursery pigs challenged with F18^+^*E. coli*. This could be related to the relatively high bioavailability of chelated minerals rather than inorganic source forms. A possible reason for this observation could be that chelated minerals reach the jejunum without interacting with other compounds, such as phytate, and the Zn ions would be free to be absorbed into intestinal epithelial cells into intestinal epithelial cells (Gupta et al., 2015). Based on previous findings, ZnGly may have a distinctive mode of action to directly support Zn homeostasis and improve the intestinal health of pigs under F18^+^*E. coli* challenge.

The desire to understand the function of the intestinal microbiota has been growing as it is highly involved in the intestinal barrier function and its interactions with commensal bacteria for host health and prevention of the invasion of opportunistic harmful bacteria (Shi et al., 2017; Duarte and Kim, 2021). According to previous studies, enterotoxigenic *E. coli* challenge can cause the imbalance of jejunal microbiota, resulting in a decreased abundance of commensal bacteria, including Firmicutes and Bacteroidetes, and an increased the abundance of opportunistic harmful bacteria such as Proteobacteria, including *Helicobacteraceae* and *Enterobacteriaceae* (Duarte et al., 2020; Xu et al., 2022). However, the results in this study show that the F18^+^*E. coli* challenge did not change the diversity and relative abundance of mucosa-associated microbiota at phylum and family levels in the jejunum of nursery pigs. According to Luise et al. (2019b), the physiological response of pigs to enterotoxigenic *E. coli* challenge has been varied, and the dosage of enterotoxigenic *E. coli* was not clearly defined among previously published articles for the oral inoculated challenge model. The results of this study indicate that the F18^+^*E. coli* challenge at 6 × 10^9^ CFU/mL may lead to less severe changes to the biological significance of the consistent impacts of F18^+^*E. coli* challenge on jejunal mucosa-associated microbiota of nursery pigs.

*Enterobacteriaceae,* a large family of Gram-negative bacteria found in the intestine of pigs, is considered opportunistic harmful bacteria and includes *Escherichia, Klebsiella, Salmonella, Shigella,* and *Citrobacter spp,* and thus *Enterobacteriaceae* have been connected to intestinal bowel diseases as these bacteria impaired intestinal barrier function and increase inflammation, oxidative stress, and crypt cell proliferation (Baldelli et al., 2021). According to Duarte and Kim (2022), enterotoxigenic *E. coli* challenge can also stimulate the overgrowth of *Enterobacteriaceae,* disrupting the balance of mucosa-associated microbiota in the intestine of pigs. The disrupted microbiota could be related to increased inflammatory response and oxidative stress (Jang et al., 2021). The results in this study show that ZnGly supplementation could reduce the abundance of *Enterobacteriaceae*, but there is limited information on how ZnGly could directly affect the Gram-negative bacteria or specific pathogenic bacteria that cause intestinal bowel diseases. Xia et al. (2021) described that Zn ions would be utilized upon pathogen invasion to produce several types of antibacterial proteins including calprotectin, psoriasin, and calgranulins, and then these antibacterial proteins lead to inactivation of superoxide defense of bacteria, inducing superoxide formation for antibacterial activity and inhibiting the growth of potential opportunistic harmful bacteria. Based on the results of this study related to changes in the expression of Zn transporters on intestinal epithelium, ZnGly is more likely to reach the intestinal mucosa tissue when compared with ZnO, leading to an effective supply of Zn ions for the modulation of jejunal mucosa-associated microbiota. Ashmead (2001) described that glycine chelation may improve the stability of metal ions possibly due to its low molecular weight among amino acids, and thus, it prevents undesirable chemical reactions in the gastrointestinal tract.

This study also shows that ZnGly supplementation increased relative abundances of Actinobacteria by increasing *Micromonosporaceae*, *Nocardiaceae, Streptomycetaceae,* and had quadratic effects on Proteobacteria through a change in relative abundances of *Alcaligenaceae*, *Methylococcaceae Pseudoalteromonadaceae,* and *Sphingomonadaceae.* These changes would be associated with microbial fermentation of two compounds, Zn and glycine, in ZnGly. Actinobacteria is a major phyla in the bacterial community and plays an important role in maintaining intestine homeostasis (Binda et al., 2018). According to Presentato et al. (2020), the microbes belonging to Actinobacteria could be tolerant or resistant to metal compounds, including Zn, within the expected concentration range due to the high mineral utilization such as biosorption, bioaccumulation, biotransformation, and metal efflux processes. Proteobacteria is also one of the most abundant amino acid fermenting bacteria in small intestine (Dai et al., 2011) and glycine would be utilized as a substrate by Proteobacteria for VFA production (Barker, 1981). Ji et al. (2022) also showed glycine supplementation at 2% could reduce the abundance of pathogenic bacteria such as *Escherichia, Shigella*, *Clostridium*, and *Burkholderiales spp.* in the intestine of nursery pigs.

Supplementation of ZnGly could not partially replace the pharmaceutical use of ZnO in the feeds without affecting any performance variables for nursery pigs challenged with F18^+^*E. coli.* According to the regression model on the BW gain of nursery pigs challenged by F18^+^*E. coli* during post challenge period, the BW gain by the pharmaceutical use of ZnO at 2,500 mg/kg would be equal to BW gain when ZnO was supplemented at 2,300 mg/kg with ZnGly at 400 mg/kg in the diet. However, ZnGly supplementation without the addition of ZnO into the feeds had similar effects on growth performance of the pigs compared with the pharmaceutical use of ZnO at 2,500 mg/kg during post challenge period. This may be because the dose response of ZnO did not rationally follow the inclusion levels. Thus, it could indicate that supplementation of ZnGly at 400 mg/kg would partially reduce the pharmaceutical use of ZnO. However, ZnO supplementation at 700 mg/kg with ZnGly at 400 mg/kg could not alleviate the negative impacts of F18^+^*E. coli* on growth performance and diarrhea of the nursery pigs. It could also indicate that the lower level of ZnO, compared with the pharmaceutical level, exacerbated the negative impacts of F18^+^*E. coli* challenge on growth of nursery pigs. However, there is limited information about the negative impacts of the low level of ZnO in nursery feeds, and previous studies have shown inconsistency in the dose response of ZnO to growth performance and other variables of interest in nursery pigs (Pieper et al., 2012; Upadhaya et al., 2018; Oh et al., 2021; Hansen et al., 2022). The pharmaceutical use of ZnO has been known as having a strong effect on preventing post weaning diarrhea and promoting the growth of nursery pigs (Kim et al., 2019), but the underlying properties or the mode of action of ZnO are still not well-determined by dose response. In general, the high level of ZnO has been known to have antimicrobial properties by inducing reactive oxygen species in the bacteria (Sharma et al., 2012). Regarding the antimicrobial properties, previous studies interestingly showed that trace amounts of antibiotics below the recommended dose could exacerbate diarrhea and systemic immune response with the imbalance of intestinal microbiota and metabolic modification of nursery pigs challenged with enterotoxigenic *E. coli* (Kim et al., 2021). Considering the antimicrobial properties, ZnO at low levels may cause intestinal microbiota and metabolic modification in nursery pigs challenged with F18^+^*E. coli*. Although this study did not investigate the metabolic conditions of the pigs, the results also show that the jejunal immune response and oxidative stress were exacerbated by ZnO supplementation at the levels lower than the pharmaceutical level with a reduction in the diversity estimates on jejunal mucosa-associated microbiota. However, it was not entirely acceptable, because these changes would be possibly associated with the ratio among the Zn sources, combinational effects, interactions with feedstuff, or relation to *E. coli* strains. Thus, future studies are needed to determine the negative impacts of lower levels of ZnO as opposed to the pharmaceutical level on the intestinal health of nursery pigs.

This study also shows that Zn glycine supplementation at 400 mg/kg could have analogous effects to pharmaceutical use of ZnO at 2,500 mg/kg on growth performance and diarrhea of the pigs, except during the first week of the post challenged period. It could also indicate that ZnGly had a different mode of action in positively affecting the intestinal health of the pigs challenged with F18^+^*E. coli* when compared with the pharmaceutical use of ZnO, especially during the first week of the post challenge period. The significant amount of unabsorbed ZnO would directly affect intestinal microbiota and inhibit the cultivation of F18^+^*E. coli* due to the improvements in growth and fecal score during the first week of the post challenge period without the changes in the expression of jejunal Zn transporters. ZnO might directly reduce the negative impacts of F18^+^*E. coli* by antimicrobial properties preventing the adhesion to the intestinal epithelium and enterotoxin productions (Fairbrother et al., 2005; Duarte and Kim, 2022). In contrast, ZnGly supplementation reduced the immune and oxidative stress responses with enhancement in the expression of Zn transporters in the jejunum of the pigs. Previous studies have also shown the positive impacts of ZnGly supplementation on Zn bioavailability, growth, and intestinal barrier function of pigs (Nitrayova et al., 2012). Thus, ZnGly would be utilized to indirectly reduce the negative impacts of F18^+^*E. coli* on intestinal health by providing high Zn bioavailability to support Zn homeostasis of intestinal epithelium for recovery from the damages sustained during the overall post challenge period.

In conclusion, F18^+^*E. coli* challenge negatively affected growth performance and the fecal score of nursery pigs by damaging intestinal villi and increasing jejunal enterocyte proliferation, inflammation, and oxidative stress. Supplementation of ZnGly at a range of 400 to 675 mg/kg could reduce the negative impacts of F18^+^*E. coli* on growth performance and diarrhea of nursery pigs. These positive changes are related to reducing jejunal inflammation and oxidative stress by enhancing jejunal Zn absorption with positive changes in jejunal mucosa-associated microbiota in the pigs. Specifically, ZnGly supplementation at 400 mg/kg could effectively reduce the use of ZnO in nursery feeds by having similar effects to the pharmaceutical use of ZnO without negatively affecting growth performance of the pigs. The main reasons for ZnGly at 400 mg/kg capable of replacing pharmaceutical use of ZnO include improved intestinal Zn absorption, reduced intestinal inflammation, and reduced oxidative stress with positive changes in mucosa-associated microbiota in the jejunum.

## Abbreviations

AA: amino acid
ADFI: average daily feed intake
ADG: average daily gain
BW: body weight
CP: crude protein
DM: dry matter
DNA: deoxyribonucleic acid
EE: ether extract
ELISA: enzyme-linked immunoassay
GE: gross energy
G:F: gain to feed ratio
STa: heat-stable toxins A
STb: heat-stable toxins B
IL-8: interleukin-8
IgA: immunoglobulin A
IgG: immunoglobulin G
MDA: malondialdehyde
NADH: nicotinamide adenine dinucleotide
OTU: operational taxonomic units
PBS: phosphate-buffered saline
RNA: Ribonucleic acid
RT–qPCR: Quantitative real-time polymerase chain reaction
SID: standard ileal digestibility
TNF-α: tumor necrosis factor alpha
VH:CD: villus height to crypt depth
ZnGly: zinc glycinate
ZnO: zinc oxide
